# Case Report: Laparoscopic Isthmocele Repair on an 8 Weeks Pregnant Uterus

**DOI:** 10.3389/fmed.2022.831588

**Published:** 2022-02-17

**Authors:** Laurentiu Pirtea, Oana Balint, Cristina Secoşan, Dorin Grigoraş, Paul Pirtea

**Affiliations:** ^1^Department of Obstetrics-Gynecology, University of Medicine and Pharmacy Timişoara, Timişoara, Romania; ^2^Clinical Emergency City Hospital, Timişoara, Romania; ^3^Hopital FOCH, Suresnes, France

**Keywords:** caesarean scar defect, niche, isthmocele, reproductive surgery, laparoscopy

## Abstract

An isthmocele, also known as a caesarean scar defect, is a long-term complication of caesarean sections with an increasing incidence. Although is often asymptomatic, it is a novel recognised cause of abnormal uterine bleeding, and it is a major risk factor for caesarean scar pregnancies or uterine ruptures in subsequent pregnancies. Currently there are no guidelines for the diagnosis and management of this condition. Several surgical techniques for the correction of isthmocele are proposed, including laparoscopic excision, vaginal repair, a combined laparoscopic-vaginal approach or more recently hysteroscopic resection. We present the case of a GII PI, 29 years old patient with a previous c-section who presented in our clinic with a positive pregnancy test for pregnancy confirmation. The ultrasound examination revealed an intrauterine evolutive 8 weeks pregnancy and a caesarean scar defect. After counselling the patient opted for pregnancy continuation and laparoscopic correction of the isthmocele. The surgery was performed under ultrasound guidance. The defect was resected, and the uterus was closed with a continuous two-layer suture. No intraoperative or postoperative complications were present. The pregnancy continued uneventfully A caesarean section was performed at term revealing a fully healed scar.

## Introduction

During the last decades, the rates of caesarean sections have increased worldwide, elevating with it the risks of complications. Among these, the isthmoceles, also known as caesarean scar defects or uterine niches are associated with a series of gynaecological and obstetrical problems (1–3). An isthmocele is defined as a triangular hypoechoic area at the site of the previous caesarean scar and represents an inadequate healing of the myometrium (4). It's reported incidence varies greatly, being comprised between 6.9 and 69% (5). Although the causes that lead to the deficient healing are not completely known, several factors have been associated with the development of the isthmoceles, including the type of incision and the closing technique (6). There are currently no standards on the evaluation or management of the isthmocele. Its presence is associated with an increased risk of obstetrical complications including morbidly adherent placenta, caesarean scar ectopic pregnancy, or uterine rupture, with a risk of 5% for large defects (7). Diagnosis of isthmoceles relies on imaging methods, but its treatment remains a controversial issue. The surgical treatment aims for the excision of the defect and restore of the endometrial thickness and include hysteroscopic, laparoscopic, or transvaginal repair procedures. Since most of the existing evidence come from retrospective case series and case reports there is not clear evidence to support the superiority of any approach.

We present the case of a caesarean scar defect diagnosed and treated by laparoscopic repair in an 8 weeks pregnant patient.

## Case Description

**Figure 1:**
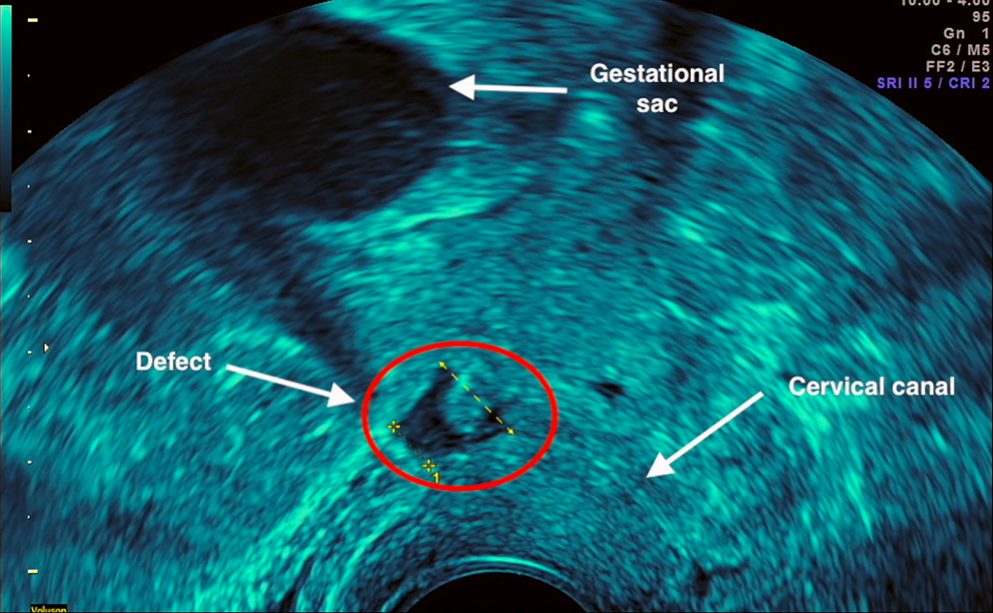
Ultrasound examination revealed an intrauterine gestational sac with alive 8 weeks embryo and a caesarean scar defect of 10 mm length involving the entire anterior lower myometrium thickness.

We report the case of a 29 years old patient, GII PI, with a previous emergency caesarean section 3 years before through a low transverse incision (2016), who presented in our service for pregnancy confirmation. The ultrasound examination revealed an intrauterine embryo, with a CRL of 20.3 mm corresponding to a 8+4 weeks pregnancy, with cardiac activity (FHR = 175 bpm) and a uterine scar defect of 10 mm length involving the entire lower anterior myometrium thickness (Figure 1). The patient was counselled regarding the risks associated with this condition and the treatment options available and their complications. She opted for surgical treatment and continuation of the pregnancy. Written informed consent for reporting the case was obtained from the patient prior to the procedure. The Institutional Review Board and Ethical Committee of “Victor Babeş” University of Medicine and Pharmacy Timişoara ruled that approval was not required for this study.

**Figure 2:**
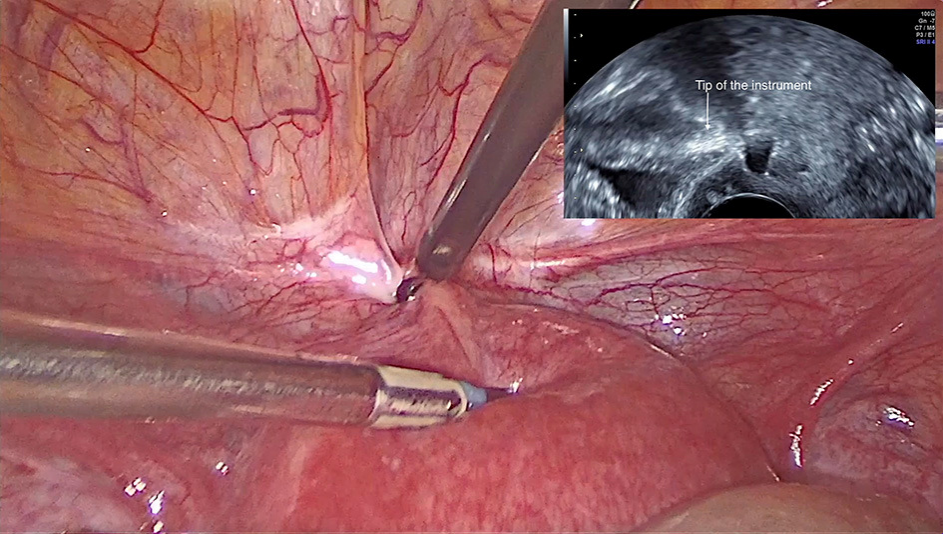
Correct identification of the scar defect using intraoperative transvaginal ultrasound guidance.

**Figure 3:**
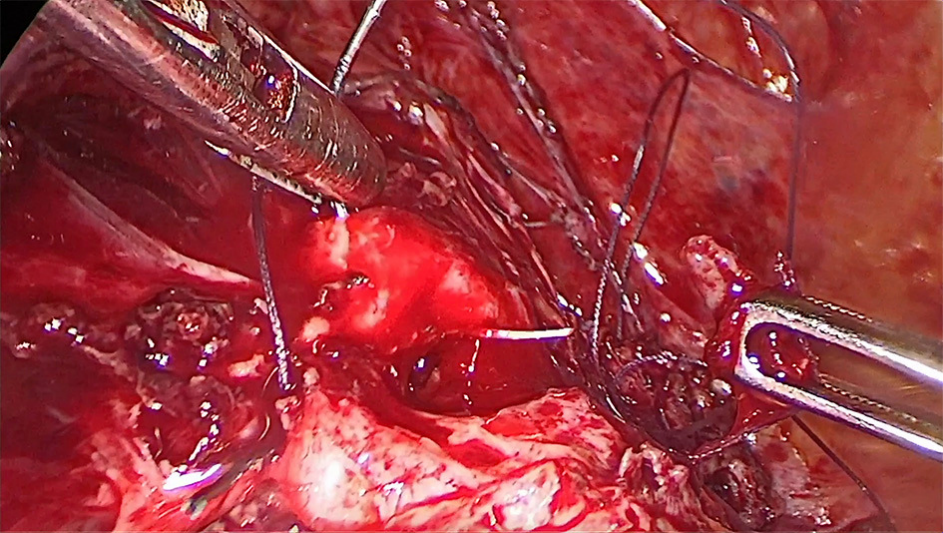
The intraoperative aspect: two-layer suture of the defect.

We performed a laparoscopic isthmocele repair on the pregnant uterus. One umbilical 10 mm optical trocar and three 5 mm trocars were used, two inserted 2 cm above and medial to the anterior superior iliac crests and the third at 5 cm below the umbilical trocar. At the peritoneal cavity inspection, we observed an enlarged uterine corpus due to the presence of the pregnancy and the urinary bladder adherent to the anterior wall of the uterus at the level of the previous caesarean section scar. We started dissection in healthy tissue at the level of the paravesical spaces bilaterally and completely mobilised the bladder. A vaginal retractor was used in order to expose the vaginal wall and was removed when the dissection was completed. Under ultrasound guidance, an area of minimal resistance was identified, corresponding to the scar defect (Figure 2). An incision at this level was created using a monopolar cautery and the margins of the defect were resected with blunt scissors into healthy tissue. The hysterotomy was sutured with slow absorbable sutures (Vicryl 2.0, Ethicon; New Jersey, USA) in two layers (Figure 3). At the end of the procedure the sutured area was evaluated by ultrasound. Also, the presence of embryonal cardiac activity was confirmed.

The duration of the surgery was 60 min with minimal blood loss (about 50 ml) and no other intraoperative or immediate postoperative complications. Twenty-four hours after surgery, the ultrasound examination revealed a myometrial thickness of 12 mm with no oedema or dehiscent spaces in the sutured area and a viable intrauterine embryo with no surrounding hematomas. The patient was discharged next day.

**Figure 4:**
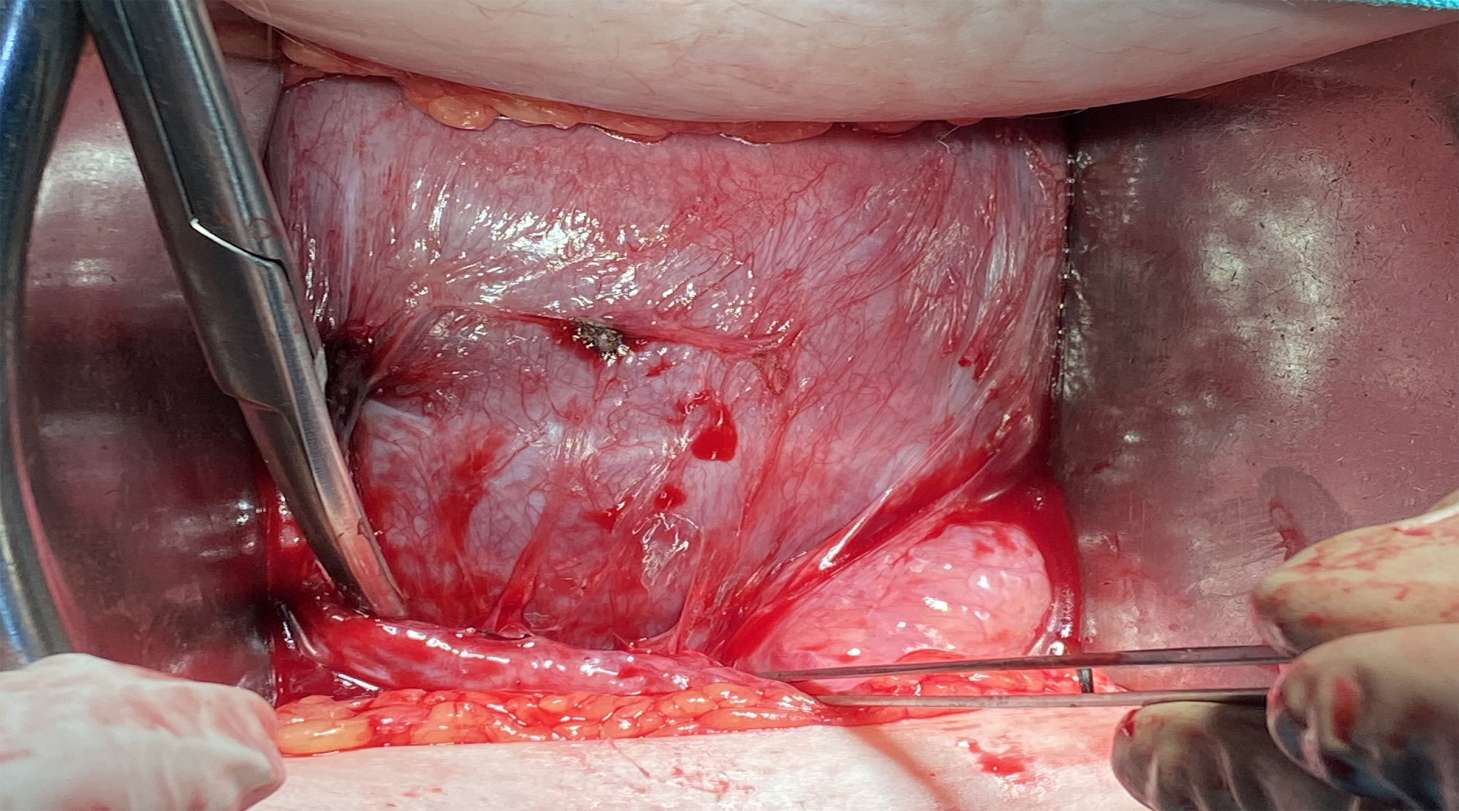
Intraoperative aspect of the lower uterine segment during the caesarean section for the current pregnancy. The lower segment is thick with a fully healed scar with no visible dehiscence.

The pregnancy continued uneventfully. A planned caesarean section was performed at 39+0 weeks of pregnancy, extracting a healthy male new-born of 3,270 g. No postoperative adhesions were present, the uterine lower segment was thick with a fully healed scar and no evidence of any dehiscence (Figure 4).

## Discussion

Currently there is no consensus for choosing the surgical approach for isthmocele treatment. However, in clinical practise two factors are used in selecting the optimal route of repair, isthmocele size and the patients desire for fertility. According to Marotta et al. an isthmocele can be classified as a large defect if the residual myometrium is >3 mm and as a small defect if the residual myometrium is <3 mm (8). For small defects, hysteroscopic resection has been reported as a safe, fast, and efficient method in controlling symptoms for the patients who did not desire fertility (9). For larger defects, hysteroscopy has been associated with an increased risk of uterine perforation and bladder injuries (10). Thus, for defect >3 mm and for the patients desiring a future pregnancy, laparoscopy is considered the optimal approach.

The laparoscopic repair technique was described by Donnez in 2008 (11). Several papers describing laparoscopic repairs have been published since, with good outcomes in reducing symptoms and improving myometrial thickness (12). In one of the largest reviews, of 38 patients, the mean post-operative thickness was 9.6 ± 1.8 mm, with no reports of complications related to the surgery and a pregnancy rate of 44%, all with term born babies (13).

In our case the patient was asymptomatic, the isthmocele being diagnosed as an incidental finding during an ultrasound examination during early pregnancy. The management of asymptomatic patient is still debatable. However, in patients desiring a future pregnancy the benefits of a surgical treatment should not be questionable. The main reason is the risk of a uterine rupture during the subsequent pregnancy, or, in our case in the current pregnancy. The existing evidence associate large defects with a high risk of uterine rupture, with a cut off value for residual endometrial thickness of <2.3 mm (14). Our patient had no residual myometrium at the caesarean scar level being thus at maximum risk for uterine rupture, foetal intrauterine demise, severe maternal bleeding, and possible compromise of the patient's future fertility. The desire to continue the pregnancy required the repair of the isthmocele.

The safety of laparoscopic surgery in pregnancy is well-known and has become the standard of care for non-obstetrical abdominal emergency condition independent of the trimester of pregnancy. Even more, in the last years, elective procedures, including laparoscopic abdominal cerclages have been performed safely in the first trimester of pregnancy.

Despite the reported safety, to avoid the risks associated with this corrective procedure during pregnancy, an ultrasound screening to identify isthmoceles and residual endometrial thickness measurement could be performed to every woman with a history of caesarean section wishing for a future pregnancy. A similar screening method is already proposed for women with a previous caesarean section that are candidates for a trial of labour (15).

One of the most crucial technical aspect of an isthmocele is the correct identification of the defect. Several techniques have been used, including illuminating the defect with the hysteroscope, or blindly leading a Hegar dilator into the defect to distend the isthmocele area. In our case both methods could not be performed due to the pregnancy. We opted to guide the identification of the defect using intraoperative transvaginal ultrasound, a method that allowed us to also visualise and restrict the surgical manoeuvres at distance from the gestational sac and to intermittently observe the embryonal cardiac activity. The myometrial thickness obtained, of 12 mm, similar to the reported mean postoperative thickness and the intraoperative aspect observed during the caesarean proved an adequate reinforcement of the myometrium.

Despite the lack of clear evidence coming from large cohort studies, the favourable outcomes reported from an increasingly number of case reports suggest that laparoscopic repair is a safe and efficient technique for isthmocele repair. However, in the asymptomatic patient the indication for surgery lies in an effort to prevent obstetrical complications during future pregnancies and in our case during the current pregnancy. Further studies are needed in order to asses different procedures and guide standardised management of caesarean scar defects in the non-pregnant and pregnant patient.

## Data Availability Statement

The raw data supporting the conclusions of this article will be made available by the authors, without undue reservation.

## Ethics Statement

Ethical approval was not provided for this study on human participants because the Institutional Review Board and Ethical Committee of Victor Babeş University of Medicine and Pharmacy Timişoara ruled that approval was not required for this study. The patients/participants provided their written informed consent to participate in this study.

## Author Contributions

LP and PP conceived and designed the study. LP and CS carried out the surgery. OB and CS analysed and interpreted the data. OB wrote the manuscript. DG critically commented on and edited the manuscript. All authors read and approved the final version of the manuscript.

## Conflict of Interest

The authors declare that the research was conducted in the absence of any commercial or financial relationships that could be construed as a potential conflict of interest.

## Publisher's Note

All claims expressed in this article are solely those of the authors and do not necessarily represent those of their affiliated organizations, or those of the publisher, the editors and the reviewers. Any product that may be evaluated in this article, or claim that may be made by its manufacturer, is not guaranteed or endorsed by the publisher.
